# Usefulness of estimated pulse wave velocity for identifying prevalent coronary heart disease: findings from a general Chinese population

**DOI:** 10.1186/s12872-022-02456-5

**Published:** 2022-01-12

**Authors:** Xiao-Wu He, Jieun Park, Wen-Sheng Huang, Li-Hua Leng, Yan Yu, Yi-Bin Pei, Gao Zhu, Shaohui Wu

**Affiliations:** 1 Department of Cardiology, The PLA Navy Anqing Hospital, Anqing, Anhui China; 2 School of Medicine, Shanghai Jiao Tong University, Shanghai, China; 3 Department of Cardiology, Anqing First People’s Hospital, Anqing, Anhui China; 4 Department of Cardiology, Shanghai Chest Hospital Affiliated To Shanghai Jiao Tong University, West Huaihai Road 241, Shanghai, China

**Keywords:** Aortic stiffness, Coronary heart disease, Estimated pulse wave velocity, Epidemiology

## Abstract

**Background:**

Aortic stiffness and coronary heart disease (CHD) share a similar spectrum of risk factors; previous studies have identified the association between aortic stiffness and CHD. Recent studies have demonstrated estimated pulse wave velocity (ePWV) as a simple and easy-acquired indicator of aortic stiffness. Our work aims to evaluate the association between ePWV and the prevalence of CHD and assess the value of ePWV for the identification of prevalent CHD.

**Methods:**

The current cross-sectional work included 7012 subjects from rural areas of southeastern China between September 2020 and February 2021. ePWV was calculated from age and mean blood pressure by specific algorithm.

**Results:**

The prevalence of CHD in our population was 3.58% (251 patients among 7012 subjects). After adjusting for age, sex, education, income and exercise level, current smoking and drinking status, body mass index, waist circumference, fasting plasma glucose, total cholesterol, high density lipoprotein, estimated glomerular filtration rate and cerebrovascular diseases, each standard deviation increment of ePWV would produce an additional 37.8% risk of prevalent CHD. Moreover, after dividing ePWV into quartiles, the 4th quartile of ePWV showed a significant risk of prevalent CHD (OR (95% CI): 3.567 (1.963–6.479)) when compared with the 1^st^ quartile. Additionally, the subgroup analysis showed the association between ePWV and prevalent CHD was robust to several common risk factors of CHD, including age, sex, body mass index, hypertension, diabetes and reduced estimated glomerular filtration rate. Finally, the area under curve (AUC) displayed an improvement when adding ePWV into common CHD risk factors (0.705 vs. 0.718. *P* = 0.044). Consistently, net reclassification index (0.436, 95% CI: 0.301–0.571, *P* < 0.001) and integrated discrimination index (0.004, 95% CI: 0.001–0.006, *P* = 0.002) demonstrated the value of ePWV to optimize the identification of prevalent CHD in the general population.

**Conclusion:**

The present analysis implicates the robust association between ePWV, a simple, rapid, and practical marker of aortic stiffness, and prevalent CHD in the general Chinese population. More importantly, the results suggest the value of ePWV as a potential marker to improve the identification of prevalent CHD.

## Introduction

Although the intensive medical therapy substantially reduces the mortality, coronary heart disease (CHD) is still one of the major causes of death worldwide. Until 2017, the worldwide mortality attributed by CHD reached 116.9 per 100,000 [1]. In China, the deaths caused by CHD in 2017 even reached 124 per 100,000 population, with a 20.6% increment when compared with the mortality of CHD in 1990 [2]. Under the pressure of this grim condition, an approach to improve and simplify CHD identification is essential to lighten the economic and healthcare burden.

Aortic stiffness is regarded as a structural and functional marker of cumulative exposure to all cardiovascular risk factors, and it acts as an arterial memory [3]. Several risk factors of aortic stiffness are also risk contributor of CHD, including hypertension, diabetes, dyslipidemia, smoking and renal disease [4]. Therefore, level of aortic stiffness may reflect the condition of coronary artery, and thereby represent the severity of CHD. Previous expert consensus has emdorsed carotid-femoral pulse wave velocity (cfPWV) as the gold-standard of aortic stiffness [5]. Furthermore, previous investigations have also revealed the incremental predictive value of cfPWV on the CHD events and related mortality [6, 7]. However, although the measurement of cfPWV is well-standardized [8], it requires expensive and specialized devices which are rarely equipped in clinical practice [9]. Accordingly, daily measurement of cfPWV to determine the severity of aortic stiffness seems impossible. Hence, there is an agreement to simplify the technology and research into inexpensive methods to measure or estimate aortic stiffness [10].

Toward this end, researchers have proposed the estimated pulse wave velocity (ePWV), which is calculated through an algorithm including age and mean blood pressure (MBP) [10]. Early research has demonstrated the strong association between ePWV and measured cfPWV [10], thereby daily measurement of ePWV can be used to monitor the severity of aortic stiffness. Moreover, previous studies have also identified the incremental predictive value of ePWV in stroke, myocardial infarction, cardiovascular mortality, etc. among several western populations [11–13]. However, limited data have focused on the association between ePWV and CHD in Chinese population, and no research has evaluated the value of ePWV in the identification of CHD. Accordingly, the current study aims to investigate the association between ePWV and prevalent CHD, and to evaluate the incremental value of ePWV for the identification of prevalent CHD in a south Chinese population.

## Methods

### Study population

**Fig. 1:**
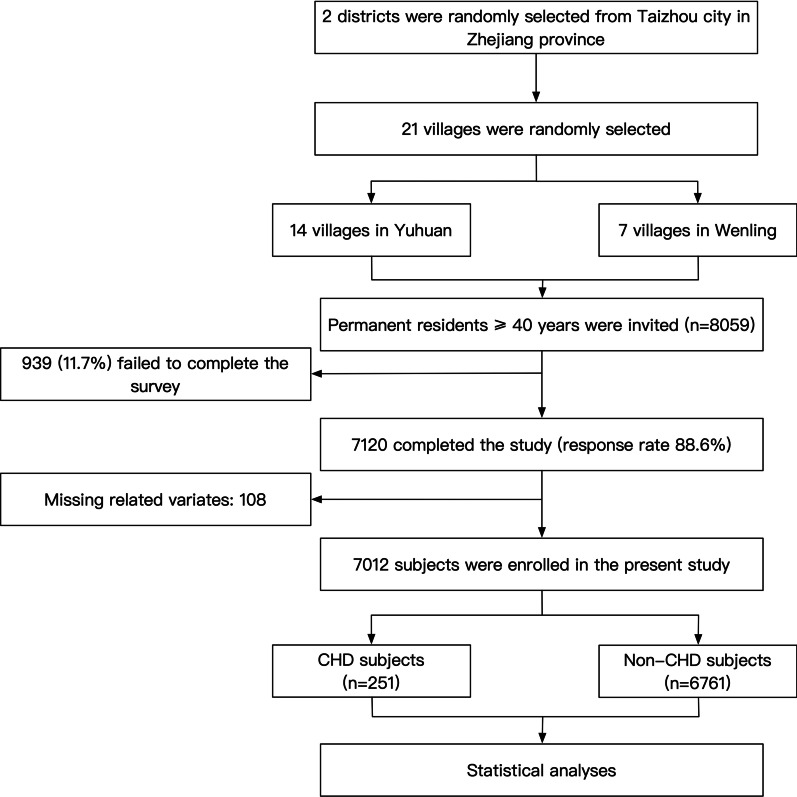
Flow chart of the enrollment process

The present work derived from a cross-sectional survey performed in the rural areas of southeastern China. The survey was conducted between September 2020 and February 2021. In order to make sure the included subjects could represent the local population, the survey adopted a multi-stage, geologically stratified and clustered random sampling approach. 21 villages from 2 districts of Taizhou city in Zhejiang province were selected randomly. Eligible subjects were defined as all permanent residents aged equal to or more than 40 years (n = 8059). Exclusion criteria included cancer, mental disorders, or pregnancy. A total of 7120 participants completed the survey. In the current analysis, we further excluded 108 participants due to censored data of co-variates, and finally enrolled 7012 subjects into statistical analyses (Fig. 1). The central ethics committee of PLA navy Anqing Hospital, the central ethics committee of Yuhuan people’s hospital and the central ethics committee of Shanghai Chest Hospital Affiliated to Shanghai Jiao Tong University approved the study protocol of the survey, and the survey was conducted in accordance with the principles of the Declaration of Helsinki. Every enrolled subject provided a written consent, if the participant was disabled, his/her relatives provided the written consent.

### Data collection and measurement

A group including neurologists, cardiologists and epidemiological specialists was employed to collect data. Before any data collection, all the crew members underwent an epidemiological course which is followed by a final test. The crew members could collect data only after passing the test. With the certification of the local health commission of Yuhuan and Wenling, the clinics in the villages were built for primary medical care. Warm and large rooms were equipped in the clinics, making the clinics met the criteria for conducting epidemiological studies. Finally, the survey employed a double-entry method for data quality control, ensuring the authenticity and accuracy of the data.

A face-to-face conversation and a standard questionnaire were conducted during a single clinical visit. The standard questionnaire collected demographic information, including age, gender, socioeconomic conditions (income and education level) and lifestyle information (exercise, smoking and exercise status). Frequent exercises were defined as moderate to heavy exercise or working intensity (sweating at cool environment) equal to or more than 3 days per week. Cerebrovascular diseases history was recorded based on subjects’ self-reports.

Anthropometric parameters were acquired when participants wear light clothes without shoes. The standard weight was acquired by a calibrated electric scale to the nearest 0.1 kg. Every participant was asked to hold in a standing position, then the standard height was acquired by a calibrated stadiometer to the nearest 0.1 cm.

Measuring of the blood pressure was conducted through calibrated electronic sphygmomanometers (HEM 907, Omron, Kyoto, Japan). The measuring process was conducted according to published guideline [14]. Step 1: Subjects were asked to avoid caffeine, exercise, and smoking for at least 30 min before any measurement, and were asked to empty his/her bladder. Measurement was performed in the large, quiet, and warm room of the clinics in the villages. Subjects were requested to relax in a sitting position for more than 5 min. Neither the subjects nor the stuff should talk during the rest period or during the measurement. The stuff would help subjects to remove all clothing covering the location of cuff placement. Step 2: The medical stuff chose right size of cuff for each subject (bladder of the cuff encircles 80% of the arm). Then subjects rested their arms on a desk, and the medical stuff posited the middle of the cuff on the subjects’ right upper arm at the level of the right atrium. Step 3: Measure the blood pressure for at least 3 times with an interval of 2 min. During the measurement, the subjects were asked to hold sitting position. Step 4: Use an average of ≥ 3 measurements to estimate the blood pressure level for each subject.

12-leads ECGs (resting, 10 s) was performed for each participant by using MAC 5500 (GE Healthcare; Little Chalfont, Buckinghamshire, UK). At least 2 cardiologists analyzed each ECG result through using magnifying glasses and calipers.

After fasting ≥ 8 h, fasting blood samples were collected in the morning. By using EDTA vacutainer tubes (Becton, Dickinson and Co., Franklin Lakes, NJ, USA), the blood samples were collected from every subject. Centrifugation was conducted in site to isolate serum from whole blood. After that, samples were stored at − 20 ℃. Finally, the samples were transported to a certified lab in the Taizhou first people’s hospital for quantitative analysis. Total cholesterol (TC), triglycerides (TG), high-density lipoprotein cholesterol (HDL-c), low-density lipoprotein cholesterol (LDL-c) and fasting plasma glucose (FPG) were quantified by a HITACHI auto-analyzer (H700, HITACHI, Tokyo, Japan). Meanwhile, the blood routine test was accomplished by a SIEMENS blood auto-analyzer (ADVIA2120i, SIEMENS, Berlin, German). To improve the data quality of the lab exam, 8% of the blood samples were randomly selected and were re-tested in the Clinical Laboratory of the health commission of Zhejiang province.

### Definitions

Body mass index (BMI) was calculated as weight (kg) divided by heigh squared (m*2). Hypertension was defined as mean systolic blood pressure (SBP) ≥ 140 mmHg and/or mean diastolic blood pressure (DBP) ≥ 90 mmHg. In addition, subjects with self-reported current use of anti-hypertensive drugs were recognized as hypertensive patients [14]. Diabetes was diagnosed as FPG equal to or more than 7 mmol/L and/or self-reported current use of anti-diabetic medication [15]. Mean blood pressure (MBP) was determined as DBP + 0.4 (SBP-DBP). ePWV was calculated from age and MBP [16]: ePWV = 9.587 − 0.402 × age + 4.560 × 10^−3^ × age^2^ − 2.621 × 10^−5^ × age^2^ × MBP + 3.176 × 10^−3^ × age × MBP − 1.832 × 10^−2^ × MBP.

Estimated glomerular filtration rate (eGFR) was calculated through CKD-EPI formula [17]. With the confirmation of computed tomography or magnetic resonance imaging, 2 independent neurologists performed the diagnosis of cerebrovascular disease [18]. The diagnosis of CHD in the current work adopted a similar strategy used in the Framingham Heart Study [19]. The diagnosis was based on in site ECG results and medical records. All medical records involving in-hospital and out-hospital cardiovascular diagnoses were copied and collected. 2 independent cardiologists reviewed the medical history and confirm the diagnosis of CHD (ICD-10 code, I 20 for angina pectoris, I 21 for acute myocardial infarction). Additionally, the cardiologists could also make a diagnosis of CHD according to the ECG conducted during the survey. Moreover, 2 members of the survey events committee reviewed the diagnosis for the 2nd time.

### Statistical analyses

Based on the distributions, categorical variables were summarized as frequency (percentage). Continuous variables were displayed as mean values (standard deviation, SD) or median (interquartile). Comparison of continuous variates between groups with and without CHD were conducted through Student’s *t* test or Mann–Whitney test according to the distribution of the variates. Disparity of categorical variates between groups were detected by employing Chi-square test. The differences of ordinal categorical variates between groups were discovered by the Rank-Sum test. The independent association between ePWV and prevalent CHD was investigated by multivariate logistic regression. The results were summarized as odds ratios (ORs) and 95% confidence intervals (95% CI). In the multivariate logistic analysis. Model 1 only adjusted demographic variate related to CAD or aortic stiffness. In model 2, we further adjusted anthropometric, laboratory, and medical history variates that were associated with CAD in univariate analysis or have been reported to associated with CAD or aortic stiffness. The ePWV distribution in quartile groups were: quartile 1: ePWV < 8.871; quartile 2: 8.871 ≤ ePWV < 10.346; quartile 3: 10.346 ≤ ePWV < 11.973; quartile 4: ePWV ≥ 11.973. In the last, receiver operating characteristic (ROC) curve, category-free net reclassification index (NRI) and integrated discrimination index (IDI) were used to exam the value of ePWV to optimize the identification of the prevalent CHD. All the statistical analyses were performed by SPSS 26.0 software (IBM, Armonk, USA), MedCalc (MedCalc Software Ltd., Ostend, Belgium), statistical software packages R (http://www.R-project.org, The R Foundation) and EmpowerStats (http://www.empowerstats.com, X&Y Solutions, Inc., Boston, MA, USA). A 2-tailed *P* value < 0.05 was regarded as statistical significance.

## Results

Data of the 7012 subjects were summarized in Table 1. Among the included participants, 251 (3.58%) subjects were diagnosed as CHD patients. Age was significantly higher in the CHD group. Gender distribution was statistically even between groups. The non-CHD group had significantly higher income level, exercise frequency and current drinking percentage. Meanwhile, the CHD group had significantly higher weight, WC, BMI, SBP, DBP, MBP, FPG, LDL-C level and lower HDL-C level when non-CHD group. Furthermore, more subjects had hypertension, diabetes, and cerebrovascular disease history in CHD group than in non-CHD group. Moreover, eGFR level was significantly lower in CHD group. Lastly, ePWV value was significantly higher in the CHD group (11.64 ± 1.93) than in the non-CHD group (10.49 ± 2.17) (*P* < 0.001).

**Table 1:** Characteristic data of participants divided by CHD

Variables	CHD (n = 251)	Non-CHD (n = 6761)	*p* value
Age (years)	64.84 ± 8.74	59.40 ± 10.50	< 0.001
Males (%)	85 (41.5)	2489 (39.8)	0.623
Education (%)			0.093
Primary school or lower	130 (63.4)	3663 (58.5)	
Middle school	49 (23.9)	1933 (30.9)	
High school or higher	26 (12.7)	665 (10.6)	
Income (CNY, %)			< 0.001
≤ 5000	118 (57.6)	2256 (36.0)	
5000–20,000	54 (26.3)	2756 (44.0)	
> 20,000	33 (16.1)	1249 (19.9)	
Frequent exercise (%)	129 (62.9)	5284 (84.4)	< 0.001
Current smoking (%)	46 (22.4)	1641 (26.2)	0.226
Current drinking (%)	43 (21.0)	1828 (29.2)	0.011
Height (cm)	158.88 ± 7.93	158.58 ± 7.96	0.681
Weight (kg)	64.13 ± 12.03	61.83 ± 11.13	0.006
WC (cm)	86.32 ± 12.19	82.70 ± 9.87	< 0.001
BMI (kg/m^2^)	25.34 ± 4.07	24.54 ± 3.77	0.003
SBP (mmHg)	150.01 ± 23.34	142.07 ± 23.12	< 0.001
DBP (mmHg)	90.19 ± 11.73	87.39 ± 11.86	< 0.001
MBP (mmHg)	114.12 ± 15.32	109.26 ± 15.32	< 0.001
FPG (mmol/L)	5.88 (5.22–6.77)	5.58 (5.13–6.18)	< 0.001
TC (mmol/L)	5.11 ± 1.29	4.98 ± 1.02	0.389
TG (mmol/L)	1.20 (0.87–1.85)	1.20 (0.85–1.75)	0.518
LDL-C (mmol/L)	1.97 (1.39–2.88)	1.65 (1.33–2.35)	< 0.001
HDL-C (mmol/L)	2.10 ± 0.84	2.33 ± 0.70	< 0.001
Hypertension (%)	160 (78.0)	3624 (57.9)	< 0.001
Diabetes (%)	51 (24.9)	761 (12.2)	< 0.001
eGFR (ml/min/1.73 m*2)	85.08 ± 15.22	92.94 ± 14.03	< 0.001
Cerebrovascular disease history (%)	38 (18.5)	401 (6.4)	< 0.001
ePWV (m/s)	11.64 ± 1.93	10.49 ± 2.17	< 0.001

Data are summarized as mean (SD), median (interquartile range), and numbers (percentage) according to their data type and distribution

CHD: Coronary heart disease; CNY: Chinese currency (1CNY = 0.15 USD); WC: waist circumstance; BMI: body mass index; SBP: systolic blood pressure; DBP: diastolic blood pressure; MBP: mean blood pressure; FPG: fasting plasma glucose; TC: total cholesterol; TG: triglyceride; LDL-C: low-density lipoprotein cholesterol; HDL-C: high-density lipoprotein cholesterol; eGFR: estimated glomerular filtration rate; ePWV: estimated pulse wave velocity

Rank-sum test or Chi-square test were conducted to compare categorical variables between groups. Mann–Whitney test or Student's *t* were employed to compare continuous data between groups

The association between ePWV and the prevalent CHD was evaluated by multivariate logistic regression and summarized in Table 2. In the crude model, each SD increase of ePWV increased the risk of prevalent CHD by 74.7%, and the top quartile of ePWV had 5.983 times risk of prevalent CHD when compared with the bottom quartile. With the adjustment of age, gender, education level, income level, exercise level, current smoking and drinking status, the OR of each SD increase of ePWV reduced to 1.492, and the top quartile of ePWV had a 4.991 times risk of prevalent CHD when compared with quartile 1. After further adjustment of BMI, WC, FPG, TC, HDL-c, eGFR and cerebrovascular diseases, each SD increase of ePWV cast a 37.8% additional risk of prevalent CHD. Meanwhile, after splitting ePWV into quartiles, the risk of prevalent CHD was 3.567 in the top quartile compared to the bottom quartile. Finally, we also observed a significant trend across the quartiles in the complete model.

**Table 2:** Multivariate logistic regression of ePWV for prevalent CHD

Variables	Odds ratio (95% CI)					
	Crude	*P* value	Model 1	*P* value	Model 2	P value
ePWV (Per 1 SD increase)	1.747 (1.510, 2.021)	< 0.001	1.492 (1.127, 1.976)	0.005	1.378 (1.037, 1.832)	0.027
Quartiles of ePWV						
Quartile 1	Reference		Reference		Reference	
Quartile 2	2.072 (1.162, 3.694)	0.014	2.101 (1.145, 3.857)	0.017	1.783(0.987, 3.221)	0.055
Quartile 3	4.450 (2.632, 7.521)	< 0.001	4.366 (2.373, 8.033)	< 0.001	3.230(1.838, 5.677)	< 0.001
Quartile 4	5.983 (3.585, 9.987)	< 0.001	4.991 (2.421, 10.292)	< 0.001	3.567(1.963, 6.479)	< 0.001
*P* for trend		< 0.001		< 0.001		< 0.001

Crude: no adjustment; Model 1: adjusted for sex, age, exercise level, annual income level, education level, current smoking, and drinking; Model 2: further adjusted for BMI, WC, FPG, TC, LDL-C, eGFR, and cerebrovascular disease history

Quartile 1: ePWV < 8.871; Quartile 2: 8.871 ≤ ePWV < 10.346; Quartile 3: 10.346 ≤ ePWV < 11.973; Quartile 4: ePWV ≥ 11.973

ePWV: estimated pulse wave velocity; CHD: coronary heart disease; CI: confidence interval; BMI: body mass index; WC: waist circumference; FPG: fasting plasma glucose; TC: total cholesterol; LDL-C: low-density lipoprotein cholesterol; eGFR: estimated glomerular filtration rate; SD: standard deviation

**Fig. 2:**
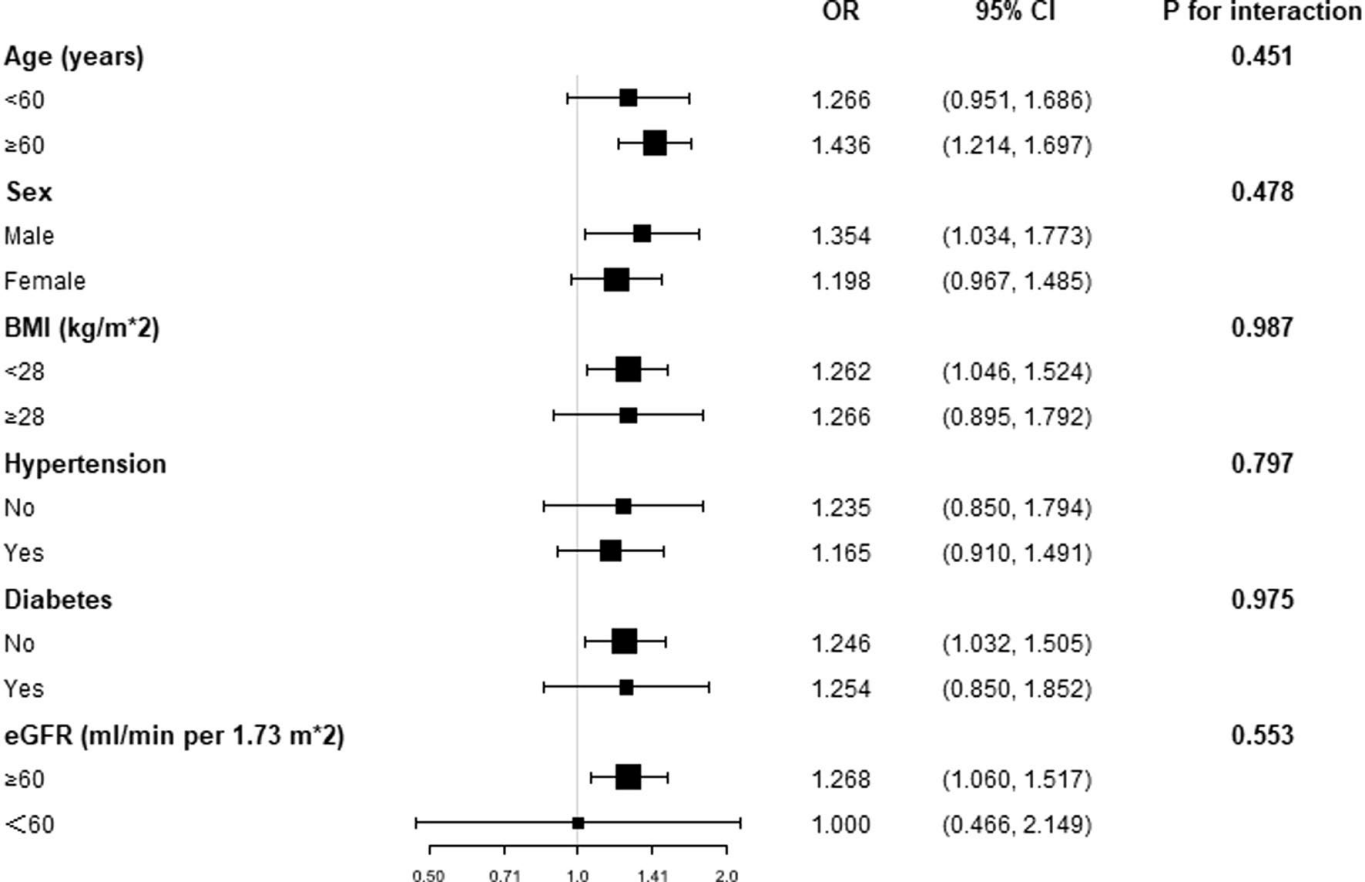
Subgroup analysis for testing the robustness of the association between ePWV and prevalent CHD. The model in each stratum was adjusted for sex, age, income level, exercise level, education level, current smoking and drinking status, BMI, WC, FPG, TC, LDL-C, eGFR, and cerebrovascular disease history except for the variate that was used to define subgroups. None of the subgroups showed significant interaction with the association between ePWV and prevalent CHD (all *P* for interaction > 0.05)

In order to evaluate robustness of the association between ePWV and prevalent CHD in specified populations, we performed stratified analyses by several common risk factors of CHD, including sex, age, BMI, diabetes, hypertension and reduced eGFR, the results were displayed in Fig. 2. In every stratum, the logistic regression model was adjusted for all covariates in the model 2 of Table 2 except the variate that was used for stratification. The results displayed that the correlation between ePWV and the prevalent CHD was steady in all strata (all *P* for interaction > 0.05). Accordingly, the correlation between ePWV and prevalent CHD was robust and might be applicable to these specified subgroups.

**Fig. 3:**
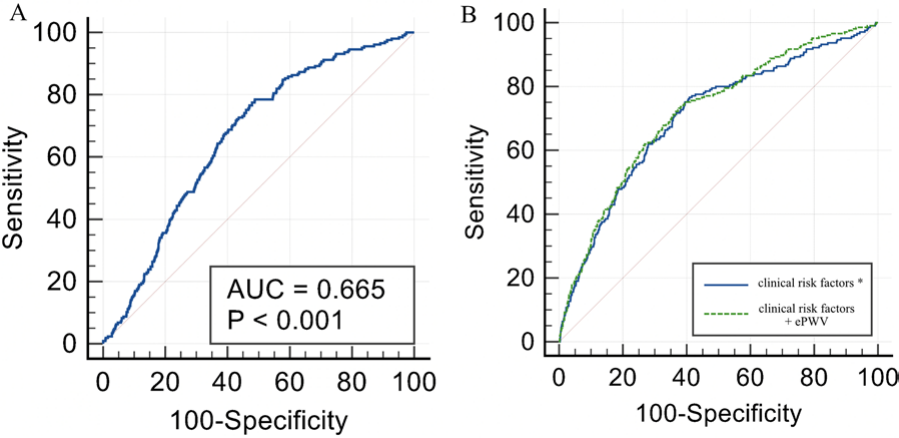
ROC analysis of the identification ability for prevalent CAD. The results of ROC analysis were displayed in Fig. 3. **a** AUC of ePWV alone for the identification of prevalent CAD, the value of AUC was 0.665 (95% CI: 0.654–0.677). **b** Comparison of clinical risk factors and clinical risk factors plus ePWV for the identification of prevalent CAD. The AUC of clinical risk factors was 0.705 (95% CI: 0.694–0.716), the AUC of clinical risk factors plus ePWV was 0.718 (95% CI: 0.707–0.729), and the *P* for comparison was 0.044. * Clinical risk factors: sex, age, current smoking and drinking status, BMI, WC, FPG, TC, LDL-C, eGFR, and cerebrovascular disease history

Finally, we employed ROC and reclassification analysis to investigate the value of ePWV to optimize the identification of prevalent CHD, the results were summarized in Table 3 and visualized in Fig. 3. The AUC of ePWV was 0.665 (95% CI: 0.654–0.677, *P* < 0.001). After introduction ePWV into several common clinical risk factors of CHD (including age, sex, current smoking and drinking status, BMI, WC, TC, HDL-C, FPG, eGFR, cerebrovascular disease), the results displayed a significant advancement of AUC (0.705 vs. 0.718, *P* = 0.044). The results of reclassification analysis showed that category-free NRI (0.436, 95% CI: 0.301–0.571, *P* < 0.001) and IDI (0.004, 95% CI: 0.001–0.006, *P* = 0.002) was significant after adding ePWV into above risk factors.

**Table 3:** ROC and reclassification analysis for ePWV to improve the identification of prevalent CHD

Model	AUC (95% CI)	*P* value	NRI (category free)	*P* value	IDI	*P* value
ePWV	0.665 (0.654, 0.677)	< 0.001				
Clinical risk factors^a^	0.705 (0.694, 0.716)	–	–	–	–	–
Clinical risk factors + ePWV	0.718 (0.707, 0.729)	0.044	0.436 (0.301, 0.571)	< 0.001	0.004 (0.001, 0.006)	0.002

^a^Clinical risk factors: sex, age, current smoking and drinking status, BMI, WC, FPG, TC, LDL-C, eGFR, and cerebrovascular disease history

ROC: Receiver operating characteristic curve; CHD: coronary heart disease; AUC: area under the curve; CI: confidence interval; NRI: net reclassification improvement; IDI: integrated discrimination index; BMI: body mass index; WC: waist circumference; FPG: fasting plasma glucose; TC: total cholesterol; LDL-C: low-density lipoprotein cholesterol; eGFR: estimated glomerular filtration rate

## Discussion

The present study evaluated the association between ePWV, a marker of aortic stiffness, and the prevalent CHD based on a general Chinese population. Our results revealed the probability of prevalent CHD increments along with the inflation of ePWV. Furthermore, our findings showed the association was robust to several major CHD risk factors, suggesting the value of ePWV in estimating prevalent CHD is applicable to these specified subgroups. Additionally, our study implicates the impact of ePWV as a cost-effective, simple, and rapid marker to improve the identification of prevalent CHD.

CHD is a major type of cardiovascular event that resulted by long-term impact of multiple cardiovascular risk factors. Previous extensive epidemiological studies have revealed a spectrum of the risk factors involved in the development of CHD, including dyslipidemia [20], hypertension [21, 22], diabetes [23, 24], obesity [25], smoking [26, 27] and etc. Meanwhile, aortic stiffness is another product of the long-term accumulation of multiple types of cardiovascular injury. The primary risk factors leading to aortic stiffness are diabetes [28, 29], blood pressure abnormalities [30, 31] and increased body fat [32, 33]. Therefore, CHD and aortic stiffness share similar etiology. Based on this, researchers evaluated the association between aortic stiffness and CHD. The results revealed the significant association between cfPWV and the risk of CHD [7]. Furthermore, researchers also demonstrated the predictive value of cfPWV for coronary events [7]. However, although the cfPWV is regarded as the gold standard for assessing aortic stiffness, its requirement of specific equipment limits its widespread use in clinical practice. Hence, there is an urgent demand for a surrogate of cfPWV to estimate the severity of aortic stiffness. The newly proposed ePWV fills the gap, previous study demonstrated the value of ePWV in estimating aortic stiffness [10]. Based on aforementioned information, we postulated that ePWV is associated with the prevalent CHD and could optimize the identification of prevalent CHD.

The findings from our analysis confirmed our hypothesis. After adjusting for demographic and clinical covariates which associated with aortic stiffness and / or CHD, the result of logistic regression still demonstrated a significant association between ePWV and the prevalent CHD, implicating ePWV could be used as an efficient indicator of the prevalent CHD. Furthermore, the subgroup analysis revealed that the positive association between ePWV and prevalent CHD was robust to age, sex, BMI, hypertension, diabetes and reduced eGFR, suggesting our results were reliable and applicable to these specified populations.

The value of ePWV for the identification of the presence of CHD was tested by ROC curve. Moreover, a significant improvement of the ability to identify prevalent CHD was also observed when adding ePWV into several common risk factors of CHD. The results from ROC analysis implicate the potential of ePWV to improve the identification of prevalent CHD. Nevertheless, although ROC analysis is the most population method for investigating the identifying ability of a new marker, it has been demonstrated to have a low sensitivity for detecting the incremental value of a new marker to identify outcomes [34]. ROC curve is unable to give appropriate information about the incremental value of a new marker to optimize the accuracy of outcome identification when adding it to established risk factors. Instead, the ROC curve can only compare the efficacy of the models with or without the new marker [35]. Based on above, statisticians proposed reclassification analysis, including IDI and NRI, to evaluate the ability of a new marker to optimize the identification of outcomes [36–38]. In summary, ROC analysis focuses on the comparison between models, while reclassification analysis pays more attention to the value of the novel markers. In the present analysis, both category-free NRI and IDI showed a significant improvement in identifying prevalent CHD when introducing ePWV into common risk factors of CHD. Therefore, both ROC curve and reclassification analysis demonstrated a significant improvement from ePWV in the identification of the presence of CHD, implicating the incremental value of ePWV in discovering the uncovered CHD in general population. By introducing ePWV to clinical practice, clinicians could identify patients with CHD more efficiently and take more corresponsive actions in the early stage of the secondary prevention of CHD.

The results of our analysis showed consistence with findings from a similar research. Ji et al. evaluated the association between ePWV and the risk of cardiovascular events in a community-based population from north China [39]. They used longitudinal data from 98,348 subjects and follow-up for 10.32 ± 2.14 years. Their results showed ePWV was associated with CVDs and all-cause mortality in their population. However, our analysis still had some significant difference with their research. First, the focus of the 2 studies is different, their research focused on the long-term predicting value of ePWV for cardiovascular events while our work payed attention to the association between ePWV and prevalent CHD and the value of ePWV to identify the uncovered CHD in the general population. Second, our population showed remarkable difference with their population, their population originated from north China while our population derived south China, the geography, economy, and lifestyle of the two area showed significant difference. According to the data, our population had substantially higher level of blood pressure, history of cerebrovascular disease, annual income, and lower rate of current drinking. Furthermore, the ePWV value in our population was higher than that in Ji’s population, implicating the level of aortic stiffness was severer in our population. The third difference derived from the outcome definition, Ji’s research set the main outcome as CVD events, including myocardial infarction, cerebral infarction and cerebral hemorhage, and all-cause death. Their outcome was a wide collection of severe cardiovascular events, not merely focused on CHD alone. Our outcome focused on CHD, and included angina pectoris, which was not defined as outcome in Ji’s research. The last innovation point of our work was the analyzing strategy. Although Ji et al. did a comprehensive analysis, they did not evaluate whether their main findings were robust in some major sub-populations. In our analysis, we observed that the association between ePWV and prevalent CHD was consistent in several major subgroups, including subgroups stratified by age, sex, BMI, hypertension, diabetes and eGFR, suggesting our main results were applicable to these specified populations.

Except for sharing similar spectrum of risk factors, aortic stiffness itself can also lead to coronary ischemia. The aging of the arterial system is charactered by structural damage, including degeneration and fragmentation of elastin, increases in collagen, thickening of the arterial wall, and progressive dilation of the arteries [40, 41]. These damage leads to a progressive stiffening of the vascular structure, further resulting in augmenting of the pressure wave velocity as it travels down the aorta. In the normal aorta, the pressure wave reflects from the periphery and returns to the heart during cardiac diastolic phase, which increase the arterial pressure during diastole and supply the coronary blood flow. When the aorta stiffens, the pressure wave velocity increases, and the reflected pressure wave arrives at the heart at systolic phase instead of diastolic phase. Hence, the coronary filling during diastolic phase is reduced and may result in coronary ischemia.

It is necessary to mentioned that there are still some limitations in our work. First, due to the cross-sectional design, the results of our analysis can only give implications for the association between ePWV and prevalent CHD, but the causality of this association still needs more prospective data to verify. Second, the prevalent rate of CHD in our population was 3.58%, relatively lower than the CHD prevalence in similar studies. However, based on the national survey of cardiovascular diseases in China, the prevalence of CHD in Chinese females and males were 0.51% and 0.74%, respectively [42]. Moreover, according to the China cardiovascular diseases report 2018, the prevalence of CHD maintained in this level in the past 10 years [43]. The national survey revealed that the prevalent rate of CHD in people aged ≥ 40 years ranged from 0.28 to 2.41% [42]. Hence, we believe the prevalence of CHD in our population is still reliable. Third, our population derived from a rural area of south-eastern China. Whether our findings are applicable to the population with different economic and geographic status still needs further research to evaluate. Last, even after adjusting for several possible confounders, bias resulted by uncollected risk factors still exist in our findings. Accordingly, research with bigger sample size and more variates are needed to confirm the association between ePWV and prevalent CHD.

## Data Availability

Data were available from the corresponding author in request if appropriate.
